# The Regulation of IGF-1 Gene Transcription and Splicing during Development and Aging

**DOI:** 10.3389/fendo.2013.00039

**Published:** 2013-03-26

**Authors:** A. M. Oberbauer

**Affiliations:** ^1^Department of Animal Science, University of CaliforniaDavis, CA, USA

**Keywords:** IGF-1, transcription, promoter, 3′-UTR

## Abstract

It is commonly known that the insulin-like growth factor-I gene contains six exons that can be differentially spliced to create multiple transcript variants. Further, there are two mutually exclusive leader exons each having multiple promoter sites that are variably used. The mature IGF-I protein derived from the multiplicity of transcripts does not differ suggesting a regulatory role for the various transcript isoforms. The variant forms possess different stabilities, binding partners, and activity indicating a pivotal role for the isoforms. Research has demonstrated differential expression of the IGF-I mRNA transcripts in response to steroids, growth hormone, and developmental cues. Many studies of different tissues have focused on assessing the presence, or putative action, of the transcript isoforms with little consideration of the transcriptional mechanisms that generate the variants or the translational use of the transcript isoforms. Control points for the latter include epigenetic regulation of splicing and promoter usage in response to development or injury, RNA binding proteins and microRNA effects on transcript stability, and preferential use of two leader exons by GH and other hormones. This review will detail the current knowledge of the mechanical, hormonal, and developmental stimuli regulating *IGF-1* promoter usage and splicing machinery used to create the variants.

For more than 60 years it has been known that the growth hormone – IGF-I axis is critical for body growth and maintenance. IGF-I is key to normal postnatal development: the absence of IGF-I is characterized by poor prenatal growth in both the rat and human (Fu et al., 2009). In addition to contributing to overall growth, the IGF-GH axis is viewed as essential for the transition of fetal to neonatal existence (Li et al., 1996). The essential role that the IGF axis plays in growth and development is further underscored in mice null for IGF-I which have significantly impaired growth while those null for IGF receptor die shortly after birth (Baker et al., 1993; Liu et al., 1993). While the general concepts of the interplay of GH and IGF-1 have been characterized, the specifics surrounding the regulation of IGF-I expression are yet to be detailed fully.

## Gene Structure

The *IGF-1* is a single gene consisting of six exons spanning nearly 90 kb of genomic DNA. The exons are alternatively spliced to generate multiple transcripts each encoding a different pre-pro-IGF-I protein possessing variable signaling peptide leader sequences. Yet after processing, all transcript isoforms give rise to same mature 70-amino acid IGF-I peptide that uses the same receptor (Holthuizen et al., 1991). The mature IGF-I peptide, conserved across species (Shavlakadze et al., 2005), contains four domains named for their similarity to those in insulin: the B-C-A-D domains. The B domain is involved in IGF-I receptor (Gauguin et al., 2008) and IGF binding protein (Magee et al., 1999) binding.

In addition to the highly conserved mature IGF-I, two other protein forms have been described in the brain (Sara et al., 1989, 1993). The two forms represent post translational cleavage of the IGF-I protein: one peptide is a 67 amino acid polypeptide lacking the first three amino terminal amino acids. It is biologically active in the brain, acts through the IGF-I receptor, and has low affinity for IGF binding proteins. The glycyl-prolyl-glutamate tripeptide cleavage product is also found in the human brain but appears to act through glutamate receptors rather than the IGF-I receptor (reviewed in Cacciatore et al., 2012).

**Figure 1:**
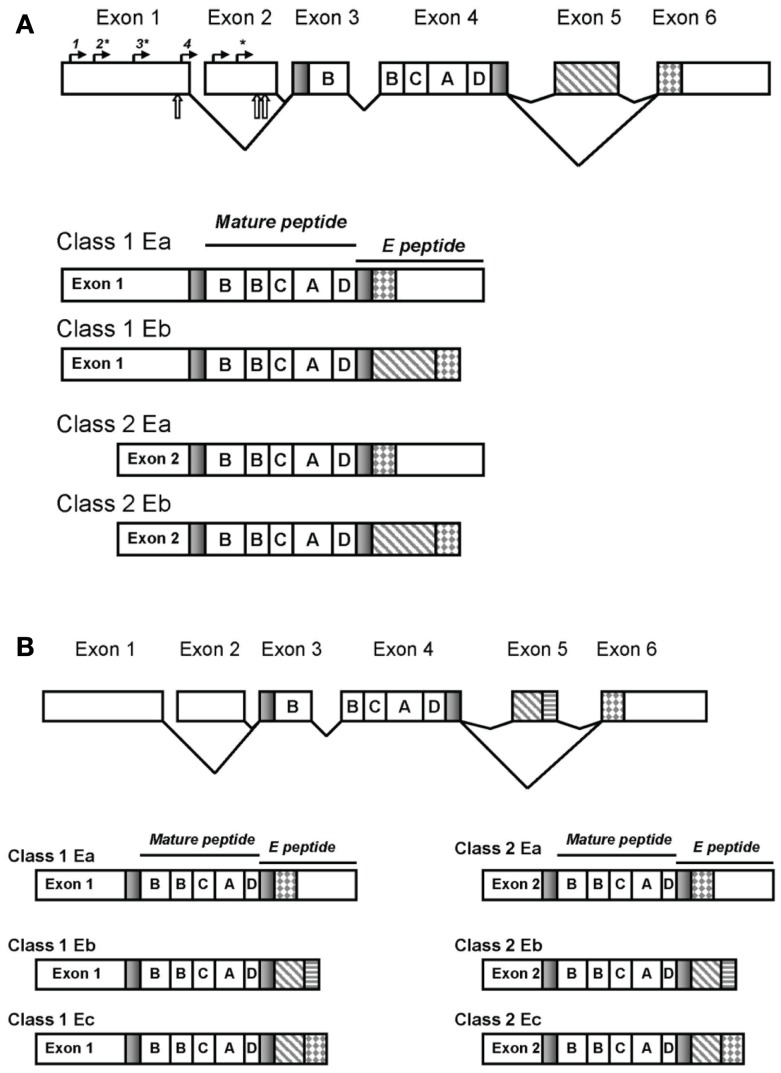
**The *IGF-1* gene structure**. Splicing and exons in the rodent gene generate distinct transcripts that vary in the 5′ and 3′ ends though the mature peptide is invariant **(A)**. Transcription initiation sites are denoted by solid arrows above the exons: sites designated 1–4 are ∼382, 343, 245, and 40 bases upstream of the 3′ end of exon 1, respectively and the two sites in exon 2 ∼70 and 50 bases upstream of the 3′ end of exon 2. Sites preferentially used are indicated with asterisks. Translation initiation is denoted with open arrows below the exons. The human IGF-I transcripts are depicted in **(B)**.

The IGF-I pre-pro peptides differ in their leader, or signal, sequences and in their carboxy-terminus. Incorporation of exon 1 or exon 2 is mutually exclusive with each serving as leader sequences of the pre-pro-IGF-I peptide; the different leader exons create different 5′UTRs (Figure 1). The pre-pro IGF-1 polypeptides undergo posttranscriptional proteolytic cleavage to remove the leader and the E-peptide carboxy-terminus giving rise to the 70-amino acid, single chain mature IGF-I.

Transcripts containing exon 1 are referred to as Class 1 transcripts whereas those containing exon 2 are referred to as Class 2 transcripts. Nearly all pre-pro peptides include 27 amino acids in the signaling peptide derived from exon 3 with the remaining signal sequences derived from the inclusion of exon 1 or 2. A minority of transcripts utilize a different transcription initiation site within exon 3 generating a shorter signaling peptide of 22 amino acids (Yang et al., 1995).

Exons 3 and 4 are invariant and encode the B, C, A, and D domains of the mature IGF peptide; exon 4 encodes two thirds of the mature IGF-I peptide. The carboxy-terminus constitutes the E-peptide domain. All E-peptides include 16 amino acids from exon 4 with variable sequences following depending upon inclusion/exclusion of exons 5 and 6. The E domain function remains unclear (Matheny et al., 2010) although much speculation surrounds this domain as it introduces the greatest variability into the IGF transcripts and has challenged consistent nomenclature. Inclusion or exclusion of exon 5 into the pre-pro IGF-I polypeptide gives rise to transcripts differing in E-peptide forms, termination codons, polyadenylation sites, and 3′UTR’s (Shimatsu and Rotwein, 1987; Shavlakadze et al., 2005).

## Splicing

In the rat, exclusion of exon 5 (that is, splicing exon 4 to exon 6) creates the Ea peptide of 35 amino acids in length. Inclusion of exon 5 (that is, splicing exon 4 to 5 and then 5 to 6) introduces 52 bases and a frame shift to generate an earlier stop codon in exon 6 thereby creating the Eb peptide of 41 amino acids; both Ea and Eb have a common 3′UTR (Shimatsu and Rotwein, 1987). In rats, the Eb transcripts account for ∼10% of IGF-I transcript expression.

In contrast, the human Eb peptide is composed of only exons 4 and 5 (Rotwein et al., 1986) whereas the splice variant containing exons 4, 5, and 6 is called the Ec peptide and is also referred to as mechanogrowth factor (MGF) (Yang et al., 1996). The human Eb splice variant is rarely detected in other species and then most notably in mice with the murine exon 3, 4, 5 splice variant existing only for class 2 IGF-I isoforms (Shavlakadze et al., 2005). The human Eb may reflect a poorly conserved 5′donor splice site in the human *IGF-1* gene causing the spliceosome to exclude exon 6 (Shavlakadze et al., 2005). The human exon 5 has a nucleolus localization sequence (Weber et al., 2000) and a polyadenylation site (Chew et al., 1995). In rodents, the Ea peptide, but not the Eb, contains two potential *N*-glycosylation sites (Roberts et al., 1987) though their role in processing and function of the IGF-I peptide remains unknown to date. Human IGF-IEa peptides possess only a single glycosylation site (Duguay et al., 1995).

An additional regulatory feature is the existence of a purine rich splicing enhancer site downstream of exon 5 that is recognized by the serine-arginine protein splicing factor-2/alternate splicing factor. Occupancy of this enhancer facilitates recruitment to the spliceosome and increases the efficiency of inclusion of exon 5 into the transcript. Importantly, the serine-arginine protein splicing factor-2/alternate splicing factor is activated by phosphorylation or by cellular localization which may mediate tissue and hormonal specific splicing of the IGF-1 transcript (Smith et al., 2002) consistent with the more limited expression of the Eb forms.

## Promoter

While there exist no classical transcription start site elements for IGF-1, the 5′UTR is highly conserved across human, rat, mouse, sheep, and cattle genomes with basal promoter activity for exon 1 in the first 412 bp of the 5′ flanking region, and negative regulatory elements beyond that (Lowe and Teasdale, 1992). In the rat exon 1 has 4 transcript start sites: ∼382 (site 1), ∼343 (site 2), ∼245 (site 3), and ∼40 bp (site 4) upstream of the 3′ end of exon 1 with sites 2 and 3 used most frequently (Adamo et al., 1991a; Simmons et al., 1993; Yang et al., 1995; Wang et al., 1997). Two additional minor start sites are found at ∼361 and ∼353 bp upstream of the 3′ end of exon 1 (Simmons et al., 1993). Usage of the four transcription sites in exon 1 generate transcripts differing in signal peptide structure. Sites 2 and 3 are upstream of a translation initiation codon and usage of either generates a 48 amino acid signal sequence (Simmons et al., 1993; Yang et al., 1995) whereas usage of site 3 is downstream of the translation initiation codon and the signal sequence derived from this site is within exon 3 and is 22 amino acids long (Yang et al., 1995). An additional exon 1 translation initiation site exists in the human gene creating a 25 amino acid signal sequence (Jansen et al., 1983).

Within the promoter region for exon 1, there exist DNAse I hypersensitive sites (Thomas et al., 1995) that were uninfluenced by GH suggesting complex transcriptional regulation of the gene. Also described in the region were consensus sequences for hepatocyte nuclear factor (HNF) transcription factor binding (Nolten et al., 1996), CCAAT/enhancer binding proteins (C/EBP) (Nolten et al., 1994), and GATA-binding protein motifs (Wang et al., 2000) all perhaps involved in basal regulation of transcription. Other enhancer elements within the promoter of exon 1 include an E box (5′-CAGCTG-3′). Binding of transcription factors, such as MyoD1, to the E box increased transcription of class 1 transcripts and is particularly important in muscle cell differentiation (McLellan et al., 2006). Recently, using computational programs, additional cis elements were predicted to lie upstream of exon 1 including sites for activator protein 1 (AP1) and interferon-consensus site binding protein/interferon-regulating factor 8; binding was then confirmed by electrophoretic mobility shift assays (Telgmann et al., 2009).

Similarly, exon 2 utilizes two main transcription start sites ∼50 and 70 bp upstream of the 3′ end of exon 2 (Adamo et al., 1991a; Simmons et al., 1993; Yang et al., 1995) with the vast majority of class 2 transcripts initiated at the site ∼50 bp upstream of the 3′ end of exon 2 (Adamo et al., 1991b). Both transcription initiation sites are upstream of the two translational initiation codons thereby generating two signal sequences of 32 or 22 amino acids although the 32 amino acid leader sequence is preferentially utilized (Yang et al., 1995).

## 3′UTR

There are four polyadenylation sites in the 3′UTR of exon 6 (Shimatsu and Rotwein, 1987) giving rise to various mRNA transcript sizes: 0.8–1.2; 1.5–2.1; 7–7.5 kb (Lund et al., 1989); the size variation predominantly arises from the differences in polyadenylation signal sequences in exon 6 (Jansen et al., 1992). The 0.8–1.2 transcript size represents either 1Ea or 2Ea and the 1.3 kb mRNA is encoded by exons 1 or 2 and exons 3, 4, 5 (in the human the 1Eb or 2Eb form). Studies of transcript stability indicate the 3′UTR following exon 6 that generated 7–7.5 kb transcripts and rich in A and U residues were less stable than the shorter 0.8–1.2 kb transcripts (Hepler et al., 1990); that is, the longest transcripts with the longest 3′UTR region have the shortest half-lives (Shimatsu and Rotwein, 1987; Lund et al., 1989). Class 2 transcripts have greater stability than class 1 (Wang et al., 2003).

Use of the polyadenylation sites in the 3′UTR affects IGF-I stability. Transcripts using the first polyadenylation site have a shorter transcript size and are more highly expressed in the liver and have greater stability (Foyt et al., 1991). Recent studies by Kawai et al. (2010) investigated the mechanism behind the reduced stability of the longer transcripts derived from the alternate polyadenylation sites. They predicted that the longer 3′UTR could provide more regulatory sites for RNA binding proteins, in particular RNA binding proteins that initiate deadenylation, one of the initial steps of RNA degradation. They determined that a gene regulated by circadian rhythm, Noc, interacts with the 3′UTR of IGF-I to destabilize the longer transcripts in a tissue-specific manner to regulate expression of the IGF-I gene product (Kawai et al., 2010).

## 3′UTR in Translation

Beyond gene transcription, the regulation of gene expression patterns occurs at translation. The regulation of translation, be it by methylation or microRNA (miRNA) repression in the 3′ UTR, is conserved across kingdoms with plants and animals using similar mechanisms. In this way, despite the conservation of sequences across species, unique and complex expression patterns can be developed (Liang and Wang, 2012).

The efficiency of IGF-I transcript translation has been described as being inversely related to the length of the 5′ UTR (Yang et al., 1995) and in rats, for class 1 transcripts, those transcripts that had initiated transcription at promoter site 4 were more efficiently translated (Yang et al., 1995). Similarly, in cattle the class 1 transcripts are translated more efficiently (fourfold) than class 2 transcripts even though there were no elements within the class 2 transcript that would be associated with reduced translational efficiency (Wang et al., 2003).

More recently the 3′UTR and its role in regulating gene expression have come under scrutiny, most especially with the conservation of translational control mechanisms across species. Bioinformatics approaches exploiting the ever expanding genome databases have enabled the interrogation of 3′UTR with the objective of identifying conserved sequences that may play a role in protein expression. For instance, binding sites for miRNAs or RNA binding proteins, generally short sequences influenced by the surrounding sequences, can govern secondary structure and possible access to the transacting factors.

A comparative analysis of the human, mouse, rat, and dog genomes uncovered 106 conserved common regulatory motifs in the 3′UTRs that are likely involved in post transcriptional regulation; a vast number of the motifs are associated with miRNAs (Xie et al., 2005). miRNAs are ∼22 nucleotide non-coding RNAs that act as post transcriptional regulators of gene expression and are found in plants and animals (Winter et al., 2009). They act to repress translation (“RNA silencing”) by binding to the 3′UTR to repress or destabilize the target transcript. Secondary structure adjacent to common miRNA sites also modulates efficiency of repression by miRNA (Liang and Wang, 2012). Approximately 25% of human miRNA genes lie in the introns of the pre-mRNA of the genes themselves; by being in the same orientation of the genes they control, they are likely processed from the introns enabling the coordination between expression of the gene and translation of the protein (Bartel, 2004, 2009). The 3′UTR of IGF-I contains 11 of the top 50 conserved miRNA sites identified by Xie et al. (2005). For example, the miRNA miR-206 preferentially targets IGF-I’s 3′UTR and miR-206 loss of function mutants exhibited accelerated growth in tilapia and increased IGF-I expression *in vivo* (Yan et al., 2013). Similarly the addition of miR-29 to cultures down regulated IGF-I in myofibroblasts (Kwiecinski et al., 2012). Two other miRNAs have been shown to interact with the 3′UTR and reduce IGF-I translation, miR-1 and miR-320, while the binding of miR-1 also reduced IGF-I stability (summarized by Lee and Gorospe, 2010).

Another element in 3′UTRs that affects stability and translatability are AU-rich elements (AREs). The protein HuR, a human ELAV-like protein, binds to the ARE sequence (loosely defined as AUUUA) (Myer et al., 1997; Brennan and Steitz, 2001) and is viewed as a critical regulator of posttranscriptional gene expression. The ARE cis element for HuR is very common in 3′-UTR’s (Gruber et al., 2011). Using a newly developed site to evaluate the presence of AREs in genes (Gruber et al., 2011), it was determined that IGF-I has 18 of the AUUUA motifs, with five showing high conservation across all species. It is notable that the IGF-I 7–7.5 kb transcripts are rich in A and U residues and have reduced stability (Hepler et al., 1990) and both IGF-I and HuR appear to be essential for normal embryonic growth and development (Katsanou et al., 2005).

As the understanding of translational regulation on gene expression expands, the role and regulatory elements within the conserved 3′UTR will assume greater importance in the overall regulation of the *IGF-1* gene.

## Biological Significance of the Isoforms

The biological significance of the isoforms remains unclear although it has long been hypothesized that the use of exon 1 use is the preferred autocrine/paracrine form (Sussenbach et al., 1992; Gilmour, 1994) while exon 2 represents the secreted endocrine form. Lending support to this is that class 2 transcripts possess a typical signal peptide motif associated with efficient secretion whereas class 1 transcripts have a longer signal peptide possibly interfering with secretion (O’Sullivan et al., 2002a), likely through myristoylation that directs the signal peptide to the endoplasmic reticulum (Temmerman et al., 2010). Additional evidence comes from class 2 transcripts being highly expressed in the liver, the primary source of circulating IGF-I (Sjögren et al., 1999; O’Sullivan et al., 2002a). Further, class 1 transcripts have a diminished capacity to bind binding proteins and are more mitogenic than the class 2 mRNAs (LeRoith and Roberts, 1991) consistent with an autocrine/paracrine role of the class 1 isoform.

Evidence blurring this categorization comes from overexpression studies. Transgenic pigs overexpressing class 1 transcripts in the muscle had elevated circulating IGF-I (Pursel et al., 2004) and cultured myoblasts overexpressing class 1 transcripts secreted IGF-I into the media (Coleman et al., 1995). Further knocking out only hepatic expression of IGF-I reduced but did not eliminate IGF-I in circulation. Most importantly, the mice null for hepatic IGF-I were not growth impaired indicating that autocrine/paracrine expression is adequate for growth promotion (Sjögren et al., 1999).

In the mouse, both the glycosylated Ea and the non-glycosylated Eb peptides facilitate binding of IGF-I to the extracellular matrix regulating bioavailability lending further evidence for an autocrine/paracrine action. The E-peptides are positively charged because of a high basic amino acid content and deglycosylation of Ea further improves its binding to the extracellular matrix by an increase in the net peptide charge (Hede et al., 2012). In the human, the pro-IGF-Eb peptide (containing exons 3, 4, 5) is not secreted but accumulates in the nucleolus suggesting an autocrine role for this isoform (Tan et al., 2002). The differential binding affinities for extracellular matrix modulated by the E-peptides has been suggested to be key in regulating secretion into circulation (Temmerman et al., 2010).

## Promoter Activation by Hormones

Even though the role of the various isoforms has not been fully elucidated, their existence suggests biological relevance. Growth hormone has been shown to directly stimulate transcription of IGF-I in rats and mice (Mathews et al., 1986; Bichell et al., 1992) and increases stability of IGF-I mRNA in sheep (O’Sullivan et al., 2002b). Hepatic class 2 expression is highly regulated by GH with GH preferentially driving exon 2 promoter usage in rats (Foyt et al., 1992; Butler et al., 1994) and in cattle (Wang et al., 2003). The regulation of class 2 transcripts by GH was seen also in lambs immunized to suppress GH secretion; these lambs with reduced GH also had significantly reduced class 2 transcripts (O’Sullivan et al., 2002a). Yet it is also known that GH can increase 1 promoter usage (Lin et al., 1998; Woelfle et al., 2003a). Cattle given a single injection of GH increased both class 1 and class 2 hepatic transcripts although class 2 transcript induction was double that detected for class 1 (Wang et al., 2003).

The well known role of GH in driving IGF-I expression has led to a dissection of the promoter region to uncover the cis-acting elements that control transcription and promoter usage in the *IGF-1* gene. As noted above, characterizing DNAse I hypersensitive sites in the exon 1 promoter failed to identify sites influenced by GH (Thomas et al., 1995). Given the high expression of class 2 transcripts in the liver, the absence of a direct GH effect on exon 1 was not unexpected.

It is now recognized that GH exerts its effects through the JAK/Stat pathway with activated Stat proteins translocating to the nucleus and serving as transcription factors. In a series of studies designed to characterize the binding of Stat proteins to the *IGF-1* promoter, the Rotwein laboratory has uncovered more than 90 potential Stat5 binding sites in the 200 kb of DNA surrounding the *IGF-1* gene for rat, human, and mouse (Chia et al., 2006). The researchers then focused on those most highly conserved sites to undertake functional studies. Two distant, paired Stat5b sites exist in the *IGF-1* rat promoter, one pair lies in the second *IGF-1* intron (the site is referred to as HS7) and the other maps 73 kb 5′ of exon 1; the tandem HS7 site was most efficient at initiating transcription in response to GH with a twofold transcription enhancement relative to the occupancy of the tandem site upstream of exon 1 (Woelfle et al., 2003a,b; Chia et al., 2006). Their results demonstrate that binding of Stat proteins, particularly Stat5b, mediates the action of GH on promoter 2 of the mouse *IGF-1*. Further, activation of HS7 site resulted in both class 1 and 2 transcripts whereas activation of the site upstream of exon 1 only induced class 1 transcripts (Chia et al., 2006). Other studies, Stat5b binding affinities were found to differ among the binding sequences, with some sequences generating reduced transcriptional activation, and that the numerous Stat5b sites interact cooperatively to modulate the transcriptional effects of GH (Varco-Merth et al., 2012). In addition to the induction of Stat5 binding, treatment with GH increases histone acetylation adjacent to each of the two *IGF-1* promoters. This chromatin modification is associated with greater accessibility of the DNA for active transcription (Rotwein, 2012).

Both exon 1 and 2 promoters are estrogen responsive. Using a chromatin immunoprecipitation (ChIP) assay, direct binding of the estrogen receptor α at a site upstream of both exon 1 and exon 2 was detected (Hewitt et al., 2010). Interestingly, although ChIP assays demonstrate direct binding of the estrogen receptor α to the human *IGF-1* promoter, there are no estrogen response element (ERE) sequences present (Sasaki et al., 2008). In ovariectomized mice treated with estrogen, class 1 transcripts were enhanced to a greater degree than class 2 indicating exon 1 was more responsive to estrogen activation (Ohtsuki et al., 2007) despite the absence of EREs. In addition to the estrogen control of promoter usage, an ERE in intron 3–4 was also seen (Hewitt et al., 2010) suggesting a potential regulatory role in splicing. Supporting that view, studies of rats and humans demonstrate that systemic exposure to steroids, androgens or estrogens, directly stimulate expression of the Ea (exons 4, 6) and MGF/Ec (exons 4, 5, 6) transcript forms in muscle (Gentile et al., 2010; Pöllänen et al., 2010) indicating a role of steroids in the expression of the different isoforms. In mice, class 1 and class 2 transcripts changed in the uterus coordinated with the estrous cycle but there were no concomitant changes in the kidney or liver. In particular, estrogen preferentially increased class 1 transcripts confirming the activity of the estrogen responsive element in the promoter of exon 1 (Ohtsuki et al., 2007). Similarly rat IGF mRNA is regulated by steroid hormones with estrogens enhancement IGF mRNA in ovarian granulosa cells and the uterus whereas dexamethasone represses IGF mRNA (Simmen, 1991).

## Tissue Expression

Most tissues are believed to use class 1 transcripts, though liver uses both and hepatic class 2 transcripts are preferentially enhanced during development (Adamo et al., 1991b; Lin et al., 1998); liver is the source tissue for approximately 75% of the IGF-I protein in circulation (Schwander et al., 1983) and in rat liver the class 1Ea is the most abundant form representing 90% of the IGF expressed (Lin and Oberbauer, 1998). Using a cre-loxP approach to create Class 2 null mice, Temmerman et al. (2010) concluded that exon 2 use in the liver is not essential for normal growth and development as the null mice showed normal development and postnatal growth with normal IGF-I serum levels. They proposed that exon 1 fully compensated for the lack of exon 2. In contrast, mice null for exon 3 were smaller and died shortly after birth – presumably due to respiratory failure (Temmerman et al., 2010) confirming the role of the mature IGF-I protein in the transition from uterine to independent existence proposed by Li et al. (1996).

Wang et al. (2003) suggest that the ubiquitous presence of both class 1 and class 2 isoforms in all mature bovine tissues evaluated (ranging from adrenal gland to brain, fat hypothalamus kidney, liver lung mammary skeletal muscle, pituitary, rumen, small intestine, spleen, and testis) indicates generic transcription factors contribute to the express of IGF-I mRNA. Their supposition is confirmed by the presence of general regulatory elements within the promoter. However, the level of expression varied with some tissues expressing the two classes differentially, again, to be expected based upon the promoter elements as discussed above. It is notable however that in contrast to the ubiquitous expression of both classes in all tissues in cattle (and sheep), the expression patterns are more restricted in humans and rats with some tissues not expressing one or the other class. Exon 2 transcripts in the rat are not expressed in some tissues such as the heart, brain, and muscle (Shemer et al., 1992). Different rat tissues appear to use different transcription initiation sites within exon 1 again primarily using the ∼343 (site 2) and ∼245 bp (site 3) upstream of the 3′ end of exon 1 though the ∼40 bp (site 4) site is constitutively used at low levels (Shemer et al., 1992; Shavlakadze et al., 2005).

Human muscle express the Ea (exons 4, 6) and MGF/Ec (exons 4, 5, 6) transcript forms (Goldspink and Yang, 2004). Specifically, the IEa and MGF/Ec/exon 4, 5, 6 transcripts are expressed in resting muscle, active muscle, damaged muscle, cardiac muscle especially post ischemia, exercised tendon, and brain again particularly in relation to ischemia; the expression in cardiac muscle and brain following ischemic insult suggests a role for MGF/Ec/exon 4, 5, 6 in the initiation of repair (reviewed in Dai et al., 2010). Interestingly, aging diminishes the response of MGF/Ec/exon 4, 5, 6 to mechanical stimulation suggesting reduced responsivity in the promoter (reviewed in Dai et al., 2010).

As noted above, MGF is the human Ec form of the rat Eb. The expression of MGF is linked to mechanical loading and stretching. The role of MGF is that of a potent promoter of muscle hypertrophy and satellite cell activation. In contrast, within muscle, Ea is implicated in proliferation and differentiation (reviewed by Philippou et al., 2007). Thus many studies have evaluated the expression and regulation of MGF in various muscle models as well as other cell models in which loading is key. For example, in osteoblasts subjected to mechanical loading, MGF expression was detected only in loaded cells and not in control cells and the response of MGF was much more rapid and transient than that of the more highly expressed IEa form (Tang et al., 2006).

## Development

There are many changes in the IGF-I transcript abundance during growth. Class 1 and class 2 increase during rat development with increases in class 1 preceding that of class 2 (Adamo et al., 1989, 1991b). During the perinatal and early postnatal developmental periods in rats, hepatic class 1 and 2 isoforms differentially changed in expression (Adamo et al., 1991b; Bichell et al., 1992; Shoba et al., 1999) and mice (Lin and Oberbauer, 1998). Expression of the IGF in developing rat liver peaked at 4 weeks postnatal declining at 6 weeks reflecting the ontogeny of the GH receptor in the liver with the proportion of class 1Ea being most abundant (Lin and Oberbauer, 1998); even though class 1Ea are more abundant, in the rat, during postnatal development and on into adulthood, exon 2 transcripts are more GH dependent (Lowe et al., 1987; Adamo et al., 1989).

Within the growth plate, class 1Ea were the most abundant transcript with high expression during the active growth phase in the proliferative and hypertrophic cell of the growth plate; by 6 weeks expression had shifted to hypertrophic cells. Class 1Eb was also expressed uniformly, albeit at low levels, across the growth plate during early growth phases but shifted to hypertrophic predominance by the late growth stage. The shift in expression corresponds to the maturation of the growth plate and demonstrates the paracrine role of 1Ea (Lin and Oberbauer, 1999). Similar to the findings of Foyt et al. (1991), the stability of class 1Ea in growth plate chondrocytes was greatly reduced relative to that of the class 1Eb or class 2Ea transcripts; the stability did not change with age indicating that stability in the growth plate was not developmentally regulated (Laugero and Oberbauer, 2000). There were however, translational differences of the transcripts with increasing age in growth plate chondrocytes (Laugero and Oberbauer, 2000).

Both *IGF-1* promoters respond to fasting, diabetes, and caloric intake with the class 2 promoter more responsive to those factors (Adamo et al., 1989, 1991b). For example, hepatic class 1 and 2 isoforms were decreased in unfed or diabetic rats (Adamo et al., 1991b). Maternal nutrient deprivation results in intrauterine growth restriction (IUGR) of the offspring. Neonates from such deprivation have deficiencies in postnatal circulating IGF-I. Additionally, the IUGR has potential long term epigenetic consequences. Neonatal rats with IUGR exhibited decreased *IGF-I* promoter 1 usage at day 0 reflecting hypermethylation of specific CpG islands (Fu et al., 2009). Although IGF-I expression was restored by day 21, the CpG islands were still hypermethylated and class 1B transcripts were still reduced (Fu et al., 2009). Promoter 2 was also hypermethylated showing reduced usage that persisted to day 21 (Fu et al., 2009). Thus, maternal nutrient deprivation can alter usage of the neonate’s *IGF-1* promoters.

Given the essential association of IGF-I and neonatal development, researchers have predicted that mutations in the *IGF-1* gene may correspond to small stature typical of IUGR. A novel splicing mutation in intron 4 of the IGF gene results in aberrant splicing and the exclusion of exon 4. The resulting truncated 1Ea transcript contains primarily the B domain sequences predicted to compete with the normal protein at the receptor potentially acting as dominant negative mutation (Fuqua et al., 2012). Individuals heterozygous for this mutation exhibit short stature.

Mutations in the polyadenylation site of the 3′UTR in exon 6 have been associated with IGF-I deficiency and with symptoms of IUGR such as short stature and neurological deficits (Bonapace et al., 2003) though other investigators have questioned the causality of the mutation in the short stature phenotype. Polymorphisms upstream of the core polyadenylation site in the 3′UTR in exon 6 were characterized in small for gestational aged children (IUGR) (Coutinho et al., 2007). The core polyadenylation site is important in correctly directing the endonucleolytic cleavage of pre-mRNA and the addition of the poly(A) tail. The investigators concluded that there exists polymorphisms and the region is critical for proper processing of the pre-mRNA, but none of the changes were causal in children born small for gestational age.

Epigenetic modification of genes, as a result of environmental perturbations or normal growth and development patterns, is known to regulate transcription. Epigenetic methylation of the *IGF-1* promoter and the 5′UTR represses transcription DNA (Fu et al., 2009). In contrast to the promoter, DNA methylation in 3′UTR increases transcription (Fu et al., 2009).

An elegant experiment in a rat model explored the role and mechanism of exon use of IGF-I in response to traumatic brain injury (Schober et al., 2012). As previously noted, inclusion of exon 5 is elevated in response to tissue insult. The investigators hypothesized that promoter usage and inclusion of exon 5 in the IGF-I transcripts reflected changes in the accessibility of the DNA. In their study, Eb was transiently elevated concomitant with a reduction of methylation surrounding the purine rich splicing enhancer site downstream of exon 5; when Eb levels decreased, methylation of the enhancer site was restored. Similarly, histone modifications were observed with the increased abundance of the Eb transcripts and those modifications were reversed when Eb transcripts were restored to normal. The promoter for exon 1 was hypermethylated in response to injury whereas the promoter for exon 2 had a histone modification profile consistent with enhanced transcription. IGF-I Eb is expressed most highly in the brain during early development after which quantities declined (Beresewicz et al., 2010) suggesting developmental regulation of the splicing machinery. The studies of Schober et al. (2012) that showed epigenetic modifications of the DNA was associated with differential promoter and exon usage lend support to this hypothesis.

Developmental regulation of the transcription factors that bind the IGF promoter also contribute to developmental IGF profiles. Croci et al. (2010) analyzed genomic sequence databases for genes having binding sites for the developmental transcription factor, early B-cell factor (EBF). They determined that IGF-I had two such binding sites, one of which (EBF-2) was highly conserved across species (mouse to man) and found in the 5′ promoter region of exon 1. Through functional studies the researchers determined that EBF highly regulates the transcription of locally produced IGF-I in the brain in a developmental fashion (Croci et al., 2010). The absence of IGF-I at defined stages of development contributes to neuronal pruning that occurs postnatal.

Yet another developmental regulatory mechanism is seen in miRNA. As noted above, the presence of miRNA binding sites in the 3′UTR are potential targets of growth regulation. For example, in tilapia, loss of function miR-206 mutants exhibited enhanced growth (Yan et al., 2013).

## Aging

Damaged or exercised muscle preferentially upregulates expression of MGF transcript form though in terms of absolute IGF isoform expression, the Ea form predominates (Hameed et al., 2004b; Hill and Goldspink, 2004). The MGF isoform is expressed in resting muscle, active muscle, damaged muscle, cardiac muscle especially post ischemia, exercised tendon, and brain again particularly in relation to ischemia. The expression following ischemic insult suggests a role for MGF in the initiation of repair (reviewed in Dai et al., 2010). Human MGF is expressed earlier than Ea during bouts of exercise (Haddad and Adams, 2002). Following muscle damage, a similar burst of MGF expression is observed which is then followed a more sustained expression of Ea (Hill and Goldspink, 2004). Mature and aged rats have a reduced capacity to express MGF when compared with young rats in a stretching and loading muscle system and the amount of MGF in aged rats reached a plateau whereas the young animals continued to rise in expression (Owino et al., 2001). MGF responds more rapidly to GH stimulation in myoblast cultures than 1Ea expression (Imanaka et al., 2008). Taken together the data indicate some mechanoresponse element in the promoter of *IGF-1* that is coordinately regulated by GH.

Muscle IGF-I also exhibits an aging response to GH. Muscle biopsies taken from young men given recombinant GH who were either exercising or sedentary expressed both class 1 and class 2 isoforms with class 1Ea predominating. In fact, for the young men, the exercise or GH regimens evinced no effect on *IGF-1* promoter usage or splicing (Aperghis et al., 2009). This differs from findings in aged men: class 1Ea increased in the muscles of elderly men given exogenous recombinant GH (Hameed et al., 2004a). The differential response is likely associated with declining GH in advancing age suggesting differential response to GH in GH-replete and GH-deficient physiological states.

Variable circulating GH impacting IGF-I isoform expression was tested in transgenic mice exposed to chronic, mildly elevated GH. These animals downregulated class 2Ea but not class 1Ea transcript expression (Lin et al., 1998). In mature mice, acute induction of high circulating GH via transgene activation resulted in rapid, transient induction of class 1Ea while class 2Ea declined. Thus, chronic low level elevated GH downregulated class 2 mRNA and when those mice were exposed to even greater GH levels class 2 was further reduced confirming that class 2 transcripts are most sensitive to GH stimulation and subject to down regulation. The persistence of class 1 transcripts to maintain responsiveness to GH despite elevated GH may reflect class 1 mediating GH’s pulsatile effects as well as being consistent with Stat5b binding to the site upstream of exon 1 driving class 1 expression.

The extensive number of miRNAs identified in posttranscriptional regulation suggests a role in aging (Inukai and Slack, 2013). The known miRNA sites in the 3′UTR of the transcript isoforms support involvement of miRNA in regulating IGF-I during aging. As evidence of this, in liver fibrosis, miRNA-29 is lost and IGF-I rises promoting collagen expression. Using cultured hepatic stellate cells to model the fibrosis arising from injury, providing miR-29 to these cultures down regulates IGF-I reducing formation of collagen (Kwiecinski et al., 2012).

## Concluding Remarks

The nuances of the various IGF-I transcripts are being slowly uncovered such as the dissection of the promoter region and the characterization of the 3′UTR. Yet, despite decades of analysis the function of some components of IGF-I transcripts remain to be discovered. The recent advances in genome wide sequencing has opened up new avenues to evaluate gene transcription and translational regulation. This will be particularly valuable to uncovering the mysteries behind the complex expression patterns seen for the *IGF-1* gene.

## Conflict of Interest Statement

The authors declare that the research was conducted in the absence of any commercial or financial relationships that could be construed as a potential conflict of interest.
